# Effect of an educational intervention based on health belief model on preventive behaviors against malaria in over 18-year-old Afghan immigrants living in Parsian

**DOI:** 10.1186/s12879-024-10016-9

**Published:** 2024-10-03

**Authors:** Zhila Pasalari, Roghayeh ezati rad, Zahra Hosseini, Habibolah Torki, Amin Ghanbarnejad, Teamur Aghamolaei

**Affiliations:** 1 Student Research Committee, Hormozgan University of Medical Sciences, Bandar Abbas, Iran; 2 Fertility and Infertility Research Center, Hormozgan University of Medical Sciences, Bandar Abbas, Iran; 3 Social Determinants in Health Promotion Research Center, Hormozgan Health Institute, Hormozgan University of Medical Sciences, Bandar Abbas, Iran; 4 Department of Health Education and Promotion, Cardiovascular Research Center, Hormozgan University of Medical Sciences, Bandar Abbas, Iran

**Keywords:** Health belief model, Preventive behaviors, Malaria disease

## Abstract

**Background:**

Malaria disease is one of the most dangerous protozoan parasitic infections with a high mortality rate in developing countries. Malaria is a public health issue, especially in Hormozgan province, and is highly affected by foreign immigrants (Pakistani and Afghani); thus, the present study aimed to evaluate the effect of an intervention based on the health belief model (HBM) on the promotion of malaria prevention behaviors in Afghani immigrants over the age of 18. The participants resided in Persian city in Hormozgan province.

**Methods:**

The present quasi-experimental study was conducted on 200 Afghans immigrants over 18 years of age who visited four comprehensive health service centers in Parsian city, south of Iran in June until December 2023. Sampling was by cluster method. In this way, the health centers were considered as clusters, and then 4 centers were randomly selected from among them (two centers of the control group and two centers of the intervention group) and participants were selected by a systematic random method by list of records in the National Integrated Health Record System (called SIB) (100participants control group, 100 participants intervention group). The data were collected using a researcher-made questionnaire based on the HBM before and after the educational intervention An educational program was designed and implemented to promote preventive behaviors against malaria in five sessions using different strategies and based on the HBM for the intervention group. The data were analyzed using independent-samples T-test, paired-samples T-test, Pearson’s correlation coefficient, analysis of covariance and linear regression. All statistical analyses and hypothesis testing were done in IBM SPSS version 25, at a significance level of 0.05.

**Results:**

In the intervention group, there was a significant difference in the mean scores of knowledge (6.48, 95% CI: 5.9,7.05), perceived susceptibility (10.57, 95% CI: 10.03, 11.1), perceived severity (16.61, 95% CI: 15.83, 16.83), perceived self-efficacy (18.26, 95% CI: **17.55**,** 18.96**), perceived benefits (15.43, 95% CI: 14.68, 16.17), perceived barriers (-22.49, 95% CI: -23.63, -21.30), cues to action (15.06, 95% CI: 14.36, 15.75), and preventive behaviors (20.05, 95% CI: 19.44, 20.65), before and after the educational intervention. *P*-value < 0.001. The regression analysis showed that the constructs of perceived susceptibility (T = 4.72, *P* < 0.001), cues to action (T = 5.30, *P* < 0.001)and perceived self-efficacy (T = 4.93, *P* < 0.001) led to the greatest change in malaria prevention behaviors(R-Square = 0.549).

**Conclusion:**

The present findings showed that the HBM -based intervention was effective in preventive behaviors against malaria in Afghans. It is recommended to design suitable educational interventions in order to increase the perceived susceptibility, cues to action and self-efficacy in order to improve preventive behaviors against malaria in Afghans.

**Supplementary Information:**

The online version contains supplementary material available at 10.1186/s12879-024-10016-9.

## Background

Malaria is the most important infectious parasitic disease and a major health issue in tropical countries of the world. This disease is transmitted to humans by the Anopheles mosquito, the cause of which is a small protozoan parasite of the genus Plasmodium, which alternately infects humans as the secondary host and the Anopheles mosquito as the primary host [1]. The symptoms of the disease include fever and chills, anemia and enlarged spleen. This disease prevails in swamps, rivers and humid areas [2]. In 2021, 247 million cases of malaria were reported worldwide. The estimated number of mortalities due to malaria in 2021 reached 619,000. More than 90% of malaria cases in the world occur in Africa in children under 5 years [3]. This region accounts for 78% of the world’s mortality rate induced by malaria [4]. The disease accounts for 30–40% of mortalities in Iran every year [5].

Hormozgan province ranks second in the rate of infection after Sistan-o-Baluchistan province. The highest percentage of Iranian cases of the disease and local transmission in the country belongs to this province [6].

**Fig. 1:**
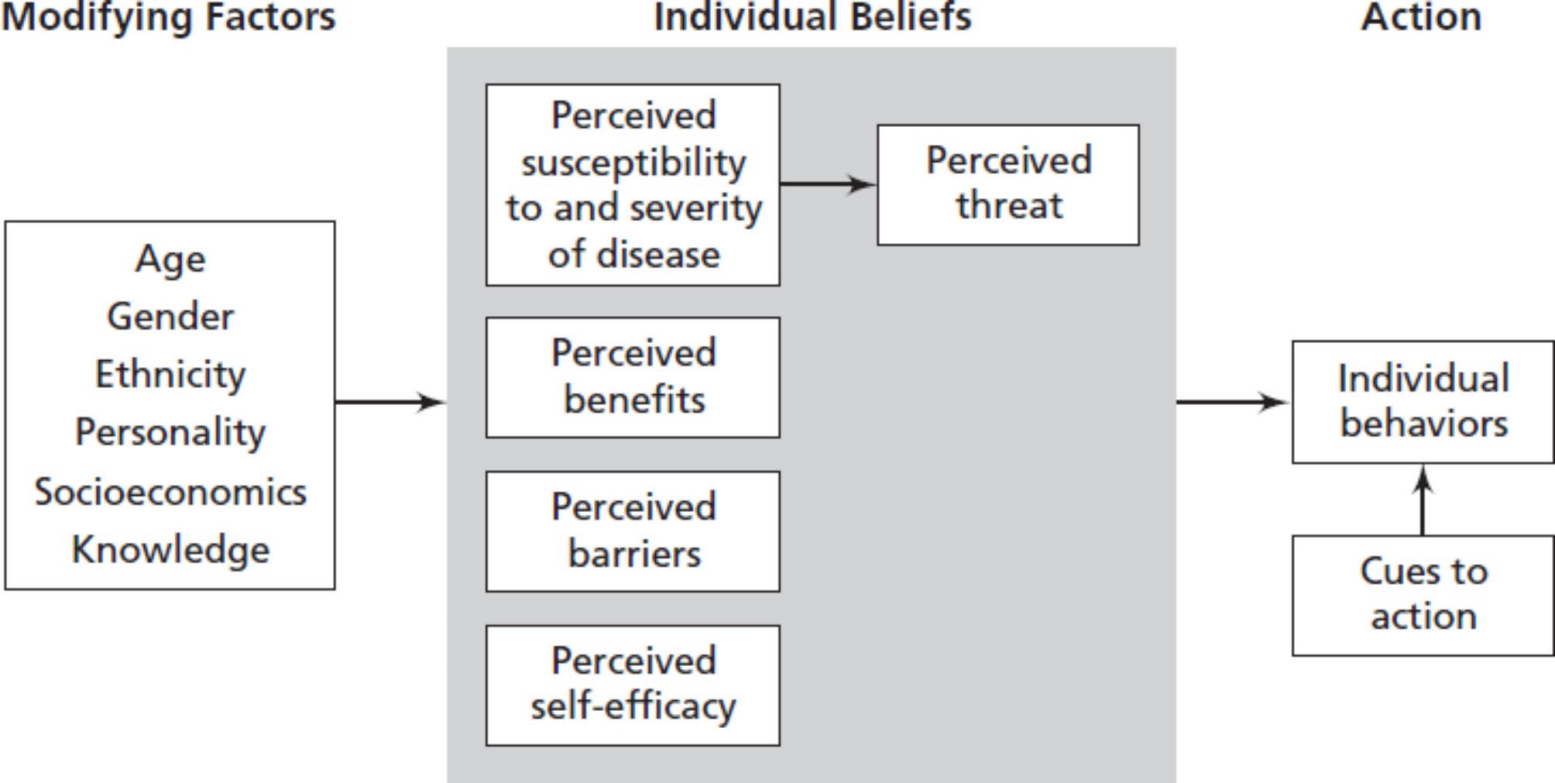
Relationship between HBM constructs

Considering the fact that until 2025, the malaria elimination program will be included in the national guidelines, teaching malaria prevention behaviors will greatly help the early diagnosis of malaria. Although there are treatments for malaria, due to the economic burden on the society, the severe pain and suffering that follows the disease, it is better to use preventive behaviors, so health education is a major priority in preventing the malaria disease [7]. An effective model in disease prevention is the health belief model (HBM), which aims to influence people’s motivation [8]. This model focuses on changing beliefs which, in turn, leads to a behavior change [9]. According to this model, if people perceive themselves susceptible to diseases such as malaria (perceived susceptibility), perceive the severity of the risk and the various complications in life (perceived severity), perceive the effect of the proposed behaviors in reducing the risk or worsening of the disease (perceived benefits) and be able to overcome the factors preventing action such as cost, time, etc. (perceived barriers) and enough confidence in their abilities to show the behavior in a way that ends effective (perceived self-efficacy), they will more likely adopt health-promoting behaviors [10] (Fig. 1).

Factors affecting the health-seeking behavior of Afaghaneh immigrants include immigration-related challenges, psychological trauma, and personal beliefs [11]. It has been suggested that health care and medical professionals should adjust their treatments to meet the cultural norms and unique health needs of immigrants [12]. Glanz et al. found that the study of perceived sensitivity and the health belief model can lead to potential solutions to improve health and change health behaviors in immigrants [13].

Studies that have used the HBM to control and prevent malaria are limited or outdated [14, 15]. No research has been conducted based on the HBM on prevention of malaria in Afghan immigrants. Parsian city in Hormozgan province has been currently planned to eliminate malaria. This city hosts Afghan immigrants, a major group at risk of malaria. These factors along with the existing weather conditions which led to the incidence of malaria for years make it necessary to make certain interventions. Therefore, the present study was conducted to explore the effect of an intervention based on the HBM on preventive behaviors against malaria in Afghan immigrants over 18 years of age living in Parsian.

## Methods

### Design and participants

The current study was a prospective quasi-experimental design that was conducted in four comprehensive healthcare centers in Parsian, a city in the west Hormozgan province in the south of Iran. The research population included all Afghan immigrants over 18 years of age visiting the comprehensive health centers of Parsian city, whose names were recorded in the National Integrated Health System (SIB). The SIB system is an integrated system for registering the health information of Iranians, in which all the data related to the health of the population are recorded during the provision of basic health services [16, 17].

Two centers were randomly selected as the intervention group (*n* = 100) and two as the control group (*n* = 100). The data were collected before the educational intervention began and then 2 months after the completion of the educational intervention in both groups.

### Sample size and sampling

The following formula was used to calculate the sample size:

$$\:n = \frac{{\begin{array}{*{20}{c}}{2 \times \:{{\left( {\:{z_{1 - \frac{{\alpha \:}}{2}}}\: + {z_{1 - \beta \:}}} \right)}^2} \times \:\bar P(1 - \bar P)\:}\\{\:\:}\end{array}}}{{\:{{(P1 - P2)}^2}}}$$

$$\:n=\frac{\begin{array}{c}2\times\:{\left(\:1.96+0.84\right)}^{2}\times\:0.34\times\:0.76\:\\\:\:\end{array}}{{\:\left(0.31\right)}^{2}}=86$$

In a similar study by Jadgal et al. [18] With a confidence interval of 95%, test power of 80%, effect size of 0.31, and the effect of the cluster sampling design, with a design effect of 2, The final sample size was estimated to be 86 participants for each group. Considering the duration of the follow-up and an attrition rate of 10%, the sample size for each group was estimated at 96 with. For more accuracy, 100 participants in each group were included in the study.

The sampling method was cluster sampling. There are seven comprehensive health centers in Parsian, which were considered as clusters, and among them, four centers (clusters) were randomly selected, which had the largest population of Afghan immigrants. Then these centers were randomly divided into intervention and control groups. In each center, 50 participants were selected to be included by a systematic sampling framework from the list of records available in the SIB system.

### Inclusion and exclusion criteria

Afghan immigrants over 18 years of age who resided in Parsian for at least 6 months and whose behavior score was less than 30% of the maximum attainable score (of the questionnaire) were included in the study. Participants with at least two sessions of absence and those who completed the questionnaires in full were excluded from the study.

### Data collection instruments

The data collection instrument was a Persian researcher-made questionnaire of four parts. The questionnaire was designed based on studies conducted and articles reviewed [19–22]. The questionnaire development was guided by a checklist from Boynton and Greenhalgh [23]The validity of this questionnaire was assessed by the content validity method. The content of the questionnaire was extracted from credible sources, including relevant books and articles. It was also approved by a panel of three experts (health education and promotion and Medical Parasitology specialists) after the necessary changes were made both qualitatively and quantitatively. The experts were asked to rate the items in terms of the words chosen, the order of presentation, and scoring. According to their feedback, revisions were made in the questionnaire. The reliability of the questionnaire was checked using the test-retest method in 20 participants of the target population, they excluded from the actual study participants. Cronbach’s alpha method was also used to check the internal correlation of the questions of the HBM constructs. The test-retest reliability was used in this research. The intraclass correlation coefficient (ICC) was estimated at 0.9 and *r* = 0.5 was significant at *p* < 0.005 level.

The first part included 8 demographic questions to enquire about age, gender, occupation, education, history of malaria, etc. The second part included questions about knowledge of malaria as a disease and its prevention methods, measured along with 11 questions with a score range of 0–11. The third part included questions about the HBM constructs. There are four questions with a score range of 4–20 to measure perceived susceptibility. The perceived severity construct included six questions with a score range of 6–30. Perceived benefits included six questions with a score range of 6–30. The perceived barriers construct included nine questions with a score range of 9–45. The perceived self-efficacy construct included six questions with a score range of 6–30. The cues to action included 6 questions with a score range of 6–30. The fourth part of the questionnaire consisted of questions on preventive behaviors against malaria with six questions and a score range of 0–24 (English language version in Supplemental File 1).

### Educational intervention

The educational content was developed based on the evaluation of the pre-test results based on the HBM in groups of 10 to 15 participants based on the interests of the participants into groups for more effective training. The research team consisted of an Medical Parasitology specialist, two general practitioners, two health care workers and two health education specialists. To develop the educational content, two face-to-face meetings were held with the members of intervention team. This educational intervention was held during 5 sessions of 60 min ( Total time of educational intervention: 300 min). In order to compile educational content, booklets prepared by the Ministry of Health were used in line with the constructs of the health belief model. The educational content and strategies selected in each session were determined based on previous studies and reliable sources [7, 21, 24] .The educational intervention was held for 35 days from July 22, 2023 to August 26, 2023, and the session s were held on a weekly basis, and the researcher allocated one day a week to the people who failed to participate in the previous training session in their group.

The sessions were held in the form of group discussions and questions and answers (Q&A). At the beginning of each session, the researcher and the participants discussed the previous session for approximately 5 min, and the comments and criticisms of the participants were used to improve other sessions.

The first session included malaria prevalence statistics in the world and Iran, knowledge of malaria disease, symptoms of the disease, ways of transmission of the disease and its prevention in order to increase the perceived susceptibility through lectures and face-to-face training. The second session included education about health hazards caused by not observing health behaviors, complications of malaria in the form of lectures, Q&A, and distribution of pamphlets to increase the severity perceived by the participants. The third session included a brief review of the contents of the first and second sessions, the benefits and importance of observing malaria preventive behaviors in the form of a group discussion to increase perceived benefits. The fourth session included a brief review of the contents of the previous sessions, presentation of a solution to remove barriers in the form of a lecture, brainstorming and group discussion in order to reduce the perceived barriers And the fifth session included a brief review of the contents of the previous sessions, questions and answers to the questions through the physician of the center, and the presentation of an educational pamphlet to the participants for cues to action, emphasizing the individual’s abilities to increase self-efficacy. Also, in the final session, each participant was given two stickers, “Malaria is preventable” and “I can do preventive behaviors”, And the participants were asked to stick the stickers on their home refrigerator to remind and adhere more to the behavior. Data collection for both intervention and control groups was simultaneous. After the implementation of the research, the prepared educational materials and content were provided to the control group in the virtual group. Then the pre-test and post-test results were compared between the two groups (intervention and control) (Table 1).

**Table 1:** Planned training sessions, activities and educational intervention strategies in the intervention group

Session #	Goal	Activities	Strategies
1	To increase perceived susceptibility	- The high prevalence of malaria in the world and Iran - Familiarity with malaria as a disease - Symptoms - Ways of disease transmission and prevention	- lecture - Face-to-face training - discussing facts and figures - Q&A
2	To increase perceived severity	- Teaching health threats caused by non-observance of health behaviors - Malaria complications - Discussing mortality facts and figures about malaria disease - Complications and consequences of the disease	- Lecture - Q&A - Pamphlet distribution
3	To increase perceived benefits	- Teaching preventive behaviors against malaria - The importance of showing preventive behaviors against malaria	- group discussion - lecture - videos
4	To decrease perceived barriers	- Exploring barriers to prevent malaria - Ways to remove perceived barriers	- lecture - brainstorming - Group discussion
5	To identify and give cues to action and increase self-efficacy	- Emphasizing one’s own abilities - Identifying and providing internal cues to action - Identifying and providing external cues to action - Dividing behavior into smaller tasks - Encouraging the use of insect repellants, door and window screens, and long-sleeved clothes at night	- Q&A with the doctor at the health center - educational pamphlet - lecture - Encouragement by successful people

### Data analysis

Mean and standard deviation were used to describe interval variables, and frequency and percentage were used for non-interval variables. Pearson’s correlation (or Spearman’s correlation) was used to test the correlation between the model constructs. Regression analysis was run to test the power of model constructs to predict the behavior. The independent-samples T-test (or Mann-Whitney U-test) were used to compare the mean scores of the model constructs in each group before and after the educational intervention. Paired-samples T-test (or Wilcoxon’s test) was used for within-group comparisons. To control the confounding effect, the multivariate covariance analysis was used. All statistical analyses and hypothesis tests were done in SPSS21, and The significance level in all tests is *P*-value < 0.05.

## Results

A total number of 200 Afghan immigrants over 18 years of age were included in this study in two groups, an intervention and a control. Most participants were female, illiterate, between 25 and 35 years of age and were housewives. The participants’ demographic information is summarized in Table 2. The findings from pearson chi-square test showed no significant difference in most demographic variables in the intervention and control groups (Table 2).

**Table 2:** Participants’ demographic information in control and intervention groups

variable	level	control		intervention		Chi-square test *P*-value
		frequency	percent	frequency	percent	
Sex	Male	25	25	24	24	**0.869**
	Female	75	75	76	76	
Education	Illiterate	70	70	71	71	**0.669**
	Elementary school	19	19	22	22	
	High school	8	8	4	4	
	University	3	3	3	3	
Age	18–27 yrs.	31	31	33	33	**0.483**
	28–37 yrs.	45	45	46	46	
	38–47 yrs.	19	19	19	19	
	48 > yrs.	5	5	2	2	
Occupation	Housewife	71	71	65	65	**0.363**
	Worker	29	29	35	35	
History of affliction with malaria	Yes	5	5	12	12	**0.076**
	No	95	95	88	88	
History of travel to Afghanistan within the past one year	yes	7	7	13	13	**0.157**
	no	93	93	87	87	
Malaria test within the past one year	yes	18	18	29	29	**0.067**
	no	82	82	71	71	
Intention to have malaria test in for the coming months	yes	18	18	29	29	0.001
	no	82	82	71	71	

non-significant *p*-value in bold

Before the educational intervention (in the pre-test), there was no significant difference between the intervention and control groups in terms of the HBM constructs and malaria preventive behaviors. However, after the educational intervention (in the post-test), a significant increase was observed in the intervention group compared to the control (Table 3).

**Table 3:** Comparison of the mean HBM constructs score between the intervention and control groups before and after intervention

variable	Group	Before intervention (Pre-test)		After intervention (Post-test)		Mean difference (95%CI Lower, Upper )	Paired-samples T-test result
		Mean	SD	Mean	SD		
Knowledge	Control	4.47	2.60	4.48	2.64	0.01 (-0.05, 0.07)	***P*** ** = 0.765**
	Intervention	4.14	2.94	10.62	0.87	6.48 (5.9,7.05)	*P* < 0.001
Perceived susceptibility	Control	6.35	2.12	6.61	2.34	0.26 (0.04, 0.47)	*P* = 0.016
	Intervention	7.70	2.75	18.27	1.82	10.57(10.03, 11.1)	*P* < 0.001
Perceived severity	Control	9.19	2.85	9.12	3.34	-0.07(-0.2, 0.34)	*P* = 0.006
	Intervention	11.69	3.50	28.30	2.68	16.61(15.83, 16.83)	*P* < 0.001
Perceived self-efficacy	Control	8.34	2.17	8.29	2.62	-0.05 (-0.34, 0.24)	*P* = 0.010
	Intervention	10.75	2.81	29.01	2.68	18.26 (17.55, 18.96)	*P* < 0.001
Perceived benefits	Control	12.32	5.05	11.43	4.23	-0.89 (-1.38, -0.39)	*P* = 0.011
	Intervention	13.02	3.86	28.45	2.32	15.43 (14.68, 16.17)	*P* < 0.001
Perceived barriers	Control	40.33	5.83	40.09	5.70	-0.24 (-0/56, 0.08)	***P*** ** = 0.137**
	Intervention	36.85	5.62	14.36	3.94	-22.49 (-23.63, -21.3)	*P* < 0.001
Cues to action	Control	10.41	3.44	10.50	3.63	0.09 (-0.2, 0.38)	***P*** ** = 0.549**
	Intervention	12.96	3.32	28.02	2.17	15.06 (14.36, 15.75)	*P* < 0.001
Malaria preventive behaviors	Control	1.34	1.18	1.51	1.11	0.17 (-0.14, 0.48)	***P*** ** = 0.291**
	Intervention	3.03	2.19	23.08	2.52	20.05 (19.44, 20.65)	*P* < 0.001

non-significant *p*-value in bold

To adjust for the effect of pre-test on the effectiveness of educational intervention on the post-test variables, covariance analysis (ANCOVA) was used. There was a statistically significant difference between the adjusted mean square of all HBM constructs in the intervention and control groups after the educational intervention. Also, the mean square of Malaria preventive behaviors after the educational intervention was different in the intervention and control groups (*p* < 0.001) (Table 4).

**Table 4:** Analysis of covariance of HBM constructs and Malaria preventive behaviors after intervention with pre-test effect controlled

variable	df	Mean square	F	*P*-value
Knowledge	1	2010.201	1010.009	< 0.001
Perceived susceptibility	1	5720.121	2907.548	< 0.001
Perceived severity	1	14410.632	4789.417	< 0.001
Perceived benefits	1	14068.141	342.632	< 0.001
Perceived barriers	1	25593.676	2058.581	< 0.001
Cues to action	1	22600.636	3344.627	< 0.001
Self-efficacy	1	850.012	486.596	< 0.001
Behavior	1	18077.135	19288.146	< 0.001

Linear regression analysis was used to test the effect of changes in HBM constructs on preventive behaviors in intervention group. The results showed that a change of one score in the perceived susceptibility construct led to an increase for 0.294 score in preventive behavior in intervention group. A change of one score the perceived benefits construct led to an increase for 0.218 in the behavior score in intervention group. A change of one score in the perceived self-efficacy construct led to an increase for 0.275 score in behavior. A change of one score un the cues to action construct led to an increase for 0.276 in malaria preventive behavior (Table 5).

**Table 5:** The results of Linear regression analysis of changes in the intervention group

Model construct	coefficient	Std. err.	Standardized coefficient	T value	*P*-value
Perceived susceptibility	0.294	0.062	0.430	4.721	< 0.001
Perceived severity	0.200	0.062	0.311	3.244	0.002
Perceived benefits	0.218	0.053	0.384	4.122	< 0.001
Perceived barriers	-0.019	0.019	-0.098	-0.979	0.165
Perceived self-efficacy	0.275	0.056	0.446	4.932	< 0.001
Cue for action	0.276	0.052	0.472	5.304	< 0.001

R-Square = 0.549

## Discussion

This study explored the effectiveness of an educational intervention based on HBM in malaria preventive behaviors in Afghan immigrants over 18 years of age living in Parsian city. The findings showed a significant difference in the mean scores of HBM constructs and preventive behaviors against malaria before and after the intervention in the intervention group. Moreover, the constructs of perceived susceptibility, cues to action and perceived self-efficacy led to the greatest change in malaria preventive behaviors.

The knowledge score in the intervention group increased significantly after the intervention, which is consistent with other studies [25–28]. Raising awareness of malaria causes a better understanding and performance of people in controlling the disease [29]. A body of research has shown that many people are not aware of the transmission of the disease by mosquitoes, while understanding the causes and ways of malaria transmission leads to appropriate preventive measures. In distant areas, people’s low level of literacy and the traditional nature of society can cause misunderstanding of the facts about malaria disease [25]. To increase awareness of health, education and maximum community participation, it is necessary to educate people by putting up malaria posters, distributing pamphlets, booklets, etc. to increase the level of community awareness [30]. In this intervention, face-to-face training sessions, lectures, group discussions, and pamphlets were used to raise people’s awareness, and the results of this study confirm the effectiveness of these methods.

As the results showed, the perceived susceptibility score in the intervention group increased significantly after the intervention, which was consistent with other studies [25, 31–33]. The perceived susceptibility construct refers to one’s susceptibility to a health issue [34]. Perceived susceptibility can be the basis for adopting preventive behaviors [35]. Changes in the perceived susceptibility construct will not occur unless people feel they are prone to a health problem. The perceived susceptibility construct can be useful in one’s intention to acquire and continue the health behavior. In the present research, a number of methods used to increase participants’ perceived susceptibility. These included lectures on malaria disease and familiarity with its symptoms and complications, ways of transmission, treatment, lectures on the role of preventive behaviors against malaria and facts and figures about malaria and the related Q&As.

In the present study, the perceived severity score in the intervention group increased significantly after the intervention, which was consistent with a body of research [25, 28, 31, 32, 36]. Perceived severity is one’s beliefs about a diseased condition and how it affects life [34]. In the current research, in order to increase the perceived severity of the disease, information was provided about the mortality rate of malaria caused by the lack of malaria preventive behaviors, complications and consequences of the disease including anemia, yellowing of the skin and eyes. To provide the required information, lectures and Q&A sessions were provided on severe malaria and its aftermath. The low level of perceived severity is the main barrier to the occurrence of preventive behaviors. Hence, more attention should be paid to perceived severity as a factor shaping a behavior that is significantly lacking in a population.

The self-efficacy score in the intervention group increased significantly after the intervention, which was consistent with other studies [36, 37] Perceived self-efficacy is one’s judgment about one’s own abilities in showing a certain behavior. It is an important component of one’s performance [38]. By removing the barriers to the behavior of interest, it is possible to increase people’s self-efficacy to show a certain health behavior [39, 40]. In the present study, to increase self-efficacy, the participants were asked to break difficult and complex tasks into smaller tasks. If it is difficult for them to use insect repellent every day, they can use it several times a week to gradually increase its use. The participants were encouraged to use insect repellants, door and window nets, and long-sleeves at night. If they used insect repellants 2–3 times a week, managed stagnant water nearby and wore long-sleeves at night, they were admired in the whole group for being successful in preventing malaria. The results showed that the afore-mentioned measures were useful to improve self-efficacy.

The present findings showed that the perceived benefit score in the intervention group increased significantly after the intervention, which was consistent with other studies [33, 41–43]. Generally speaking, to adopt a preventive behavior, one should perceive one’s benefits in developing a behavior that is beneficial, feasible and effective [25]. In the current research, lectures and video screenings were used about the benefits of malaria prevention behaviors, group discussion of malaria preventive behaviors. The results of data analysis showed that these strategies improve the participants’ perceived benefits in the intervention group.

As the findings showed, the perceived barriers score in the intervention group increased significantly after the intervention, which was consistent with other studies [25, 32, 44]. In a study conducted by Ghahrani et al., the participants believed that the cost of purchasing mosquito nets is very low compared to the protection they provided against malaria, and their willingness to use mosquito nets increased [36]. The low awareness and economic status are among the barriers to the lack of preventive behaviors. In the current study, brainstorming and group discussion were used as the perceived barriers to preventive behaviors against malaria, and the results of the data analysis showed that these strategies were effective in improving the perceived barriers in HBM to promote malaria preventive behaviors.

The cues to action score in the intervention group increased significantly after the intervention, which was consistent with several studies [25, 32, 36, 37]. The cues to action are internal or external forces that are necessary for a person to engage in an action [45]. As the results showed, to adopt preventive behaviors against malaria, the participants first felt to be susceptible to the risk factors of the living condition, and then they were reminded of the severity of risk and the seriousness of its various complications in economic, psychological, physical, and social dimensions. Thus, they began to perceive the useful and feasible nature of the educational program, learn about and control the existing barriers with the right prevention and management strategies. In the current research, a pamphlet was used about malaria (definition, and symptoms of the disease, etc.) to act as a cue for action. Doctors and health care workers were told about the prevention of malaria and gave a speech accordingly because the participants were better influenced by doctors and health workers. These cues to action were external in type. Also, increasing the perceived susceptibility of family, friends and acquaintances had a great impact on the participants.

As the present study showed, the preventive behaviors score in the intervention group increased significantly after the intervention, which was consistent with a body of research [25, 32, 36, 37]. The findings showed that the HBM constructs managed to improve malaria prevention behaviors. Health behavior is the most basic goal of health education, and health education will not be complete without creating healthy and principled health behavior [46].

In the present study, an integrated approach was used as a strategy to develop preventive behaviors against malaria to reduce the incidence rate of the disease. This strategy involved the use of several methods to prevent malaria. Malaria prevention methods in this approach include cutting down on the food sources and breeding places for mosquitoes (e.g., drying the water spots near the house, stagnant water, and cleaning the garbage around the house), protecting oneself and the family (e.g., wearing long-sleeves, using mosquito nets, and mosquito repellent cream), and blocking the entry of mosquitoes into the house (e.g., proper ventilation, use of nets on doors and windows, closing doors and windows early at night). These methods were used to reduce the mosquito population and prevent mosquito bites. It has been emphasized in many studies that the most important strategy to prevent malaria is to prevent mosquito bites [47, 48].

In order to retain the participants for the intervention program, various actions were taken, including: involving participants in all stages of the program and increasing their self-efficacy, telephone calls once a week to encourage and monitor, encouraging successful participants to perform preventive behaviors in sessions, creating motivational commitment in the group to achieve the goal and asking families to motivate and support the participants to perform preventive behaviors, which can help researchers in conducting similar research in the future.

Finally, it should be noted that the educational intervention based on the HBM had a positive effect on the prevention of malaria.

One limitation of this study is the absence of some participants in educational sessions for different reasons such as part-time work, lack of boredom, etc. In these cases, this problem was largely resolved with frequent follow-ups. Moreover, the differences in participants’ literacy level and A large number of questionnaire questions are the other limitations of this study. Caution should be made in comparing these findings with other studies. Another limitation of the study was the random sampling, which may suffer from selection bias, which was tried to be controlled by carefully considering and controlling the Inclusion and exclusion criteria, as well as the statistical methods such as Linear regression. In order to control the contamination bias, we tried to select the intervention and control groups from areas that are far away and it is not possible to exchange information, and the steps of the study were carried out according to the designed study protocol.

## Conclusion

The findings showed that after the educational intervention, all the HBM constructs in the intervention group had a significant positive change. Based on the results of this study, it is recommended to consider increasing perceived susceptibility, cues to action and self-efficacy with appropriate educational strategies and resources in order to improve preventive behaviors against malaria in Afghans. It is also recommended to empower health care workers to provide practical solutions in the field of malaria prevention and control based on the health belief model and prepare a summary and comprehensive Educational contents on the prevention of malaria and show it to people at risk. It is suggested that researchers use the health belief model in the future to prevent malaria in other groups at risk, and in diseases similar to malaria, also in the form of a mixed model with other effective models in this field.

## Electronic supplementary material

Below is the link to the electronic supplementary material.

Supplementary Material 1

## Data Availability

The datasets used and/or analyzed during the current study are available from the corresponding author on reasonable request.

## Abbreviations

HBM: Health belief model

## References

[1] Malaria RB. World malaria report 2005. World Health Organization and UNICEF; 2005.

[2] Tuteja R. Malaria – an overview. FEBS J. 2007;274(18):4670–9. 17824953 10.1111/j.1742-4658.2007.05997.x

[3] Abbasi E, Vahedi M, Bagheri M, Gholizadeh S, Alipour H, Moemenbellah-Fard MD. Monitoring of synthetic insecticides resistance and mechanisms among malaria vector mosquitoes in Iran: a systematic review. Heliyon. 2022. 10.1016/j.heliyon.2022.e08830 PMC8808063 35128113

[4] Najafi-Sharjabad F, Darabi AH, Kazerouni A, Rayani M. Epidemiological features of malaria in Bushehr province, southwest of Iran. Ann Parasitol. 2022;68:93–101. 35491835 10.17420/ap6801.413

[5] Nodez SMM, Khosravani M, Rafatpanah A, Jafarpour S, Jaberhashemi SA. Imported malaria and epidemiologic components of this infection in Qeshm Island, Iran. Int J Mosq Res. 2018;5(1):9–143.

[6] Iwarsson S, Ståhl A. Accessibility, usability and universal design—positioning and definition of concepts describing person-environment relationships. Disabil Rehabil. 2003;25(2):57–66. 12554380 10.1080/dre.25.2.57.66

[7] Glanz K, Rimer BK, Viswanath K. Health behavior and health education: theory, research, and practice. Wiley; 2008.

[8] Strecher VJ, Champion VL, Rosenstock IM. The health belief model and health behavior. 1997.

[9] Sheeran P, Abraham C. The health belief model. Predicting Health Behav. 1996;2:29–80.

[10] Champion VL, Skinner CS. The health belief model. Health behavior and health education: Theory, research, and practice. 2008;4:45–65.

[11] Ahmadinia H, Heinström J, Eriksson-Backa K, Nikou S. Health susceptibility perceptions among Iranian, Afghan and Tajik minorities in three nordic countries. Int J Migration Health Social Care. 2024;20(2):290–304.

[12] Mölsä M, Tiilikainen M, Punamäki R-L. Usage of healthcare services and preference for mental healthcare among older Somali immigrants in Finland. Ethn Health. 2019;24(6):607–22. 28669226 10.1080/13557858.2017.1346182

[13] Glanz K, Rimer BK, Viswanath K. Theory, research, and practice in health behavior and health education. 2008.

[14] Kalantari M, Soltani Z, Ebrahimi M, Yousefi M, Amin M, Shafiei A, et al. Monitoring of Plasmodium infection in humans and potential vectors of malaria in a newly emerged focus in southern Iran. Pathogens Global Health. 2017;111(1):49–55. 28078947 10.1080/20477724.2016.1271094 PMC5375612

[15] Basseri H, Holakouie Naieni K, Raeisi A, Shahandeh K, Akbarzadeh K, Ranjbar M, et al. Comparison of knowledge, attitude and practice (KAP) regarding malaria transmission and protection between Afghan refugees and Iranian residents in Iranshahr, 2005–2006. Iran J Epidemiol. 2008;3(3):7–13.

[16] Bitaraf S, Janani L, Hajebi A, Motevalian SA. Information System Success of the Iranian Integrated Health Record System based on the clinical information System Success Model. Med J Islam Repub Iran. 2022;36:25. 35999915 10.47176/mjiri.36.25 PMC9386750

[17] Rangraz Jeddi F, Nabovati E, Bigham R, Khajouei R. Usability evaluation of a comprehensive national health information system: relationship of quality components to users’ characteristics. Int J Med Inf. 2020;133:104026. 10.1016/j.ijmedinf.2019.104026 31733603

[18] Jadgal KM, Zareban I, Rakhshani F, Shahrakipour M, Sepehrvand B, Alizadeh Sivaki H. The effect of health education according to the theory of planned behavior on malaria preventive behavior in rural men of Chabahar. J Res Health. 2012;2(2):237–46.

[19] Ghodsi M, Maheri M, Joveini H, Rakhshani MH, Mehri A. Designing and evaluating Educational intervention to improve preventive behavior against cutaneous leishmaniasis in endemic areas in Iran. Osong Public Health Res Perspect. 2019;10(4):253–62. 31497498 10.24171/j.phrp.2019.10.4.09 PMC6711717

[20] Cha H, Lee K. Development of health belief in emerging infectious respiratory disease preventive behaviors’ scale. Nurs Health Sci. 2022;24(2):508–18. 35510531 10.1111/nhs.12948

[21] Nasirzadeh M, Kaveh F, Sayadi AR, Asadpour M. Effect of educational intervention on preventive behaviors of brucellosis among health volunteers in Rafsanjan city: application of health belief model. J Educ Health Promot. 2021;10:369. 34912905 10.4103/jehp.jehp_1256_20 PMC8641756

[22] Ghahremani L, Azizi M, Moemenbellah-Fard MD, Ghaem H. Malaria preventive behaviors among housewives in suburbs of Bandar-Abbas City, south of Iran: interventional design based on PRECEDE model. Pathog Glob Health. 2019;113(1):32–8. 30784362 10.1080/20477724.2019.1583847 PMC6427622

[23] Boynton PM, Greenhalgh T. Selecting, designing, and developing your questionnaire. BMJ. 2004;328(7451):1312–5. 15166072 10.1136/bmj.328.7451.1312 PMC420179

[24] Hosseini Z, Najafi P, Mohseni S, Aghamolaei T, Dadipoor S. The effect of a theory-based educational program on southern Iranian prisoners’ HIV preventive behaviors: a quasi-experimental research. BMC Public Health. 2022;22(1):1342. 35836148 10.1186/s12889-022-13763-z PMC9281156

[25] Hosseini M. Design and evaluation the effect of educational intervention based on protection motivation theory on preventive behavior of malaria among Mulavis, in the Sarbaz city. Tarbiat Modares University; 2020.

[26] Ayi I, Nonaka D, Adjovu JK, Hanafusa S, Jimba M, Bosompem KM, et al. School-based participatory health education for malaria control in Ghana: engaging children as health messengers. Malar J. 2010;9(1):1–12. 20398416 10.1186/1475-2875-9-98 PMC2865503

[27] Diouf G, Xu Y, Kpanyen PN, Cai L, Gan X, Nie S. Evaluation of the effectiveness of intervention for malaria control in rural areas in China. Global J Health Sci. 2010;2(2):41.

[28] Rostami A. Examining the application of the educational model of health belief on preventive behaviors against malaria. Tarbiat Modares University; 1999.

[29] Marsh V, Kachur S. Malaria Home Care and Management. Policy to Strategy and Implementation Series, trial issue, series ed Mehra S London and Liverpool: Malaria Consortium. 2002.

[30] Bista MB. The annual internal assessment of malaria and kala-azar control activities, 2003: HMG, Ministry of Health, Department of Health Services, Epidemiology ‖; 2005.

[31] Suwannachat J. An application of the Protection Motivation Theory with Social Support to promote Malaria Prevantion behaviors among High Risk groups in Klonghad District. Sakaeo Province: Mahidol University; 2006.

[32] Rakhshani F, Ansari-Moghadam A, Mohammadi M, Ranjbar M, Raeisi A, Rakhshani T. Community perceptions and practices about malaria prevention and control in Iran. Iran J Public Health. 2014;43(1):62. 26060681 PMC4454025

[33] Sadegh M. Investigating the effect of training based on the health belief model on preventive behaviors against covid-19 in pregnant mothers of Aliguderz city. Arak University of Medical Sciences and Health Services; 2023.

[34] Naser M. Comprehensive curriculum of health education volume one. Familiarity with practical concepts. Women’s Stud Res Inst. 2006;1:300–9.

[35] Ferguson WJ, Lemay CA, Hargraves JL, Gorodetsky T, Calista J. Developing community health worker diabetes training. Health Educ Res. 2012;27(4):755–65. 21926065 10.1093/her/cyr080

[36] Ghahremani L, Faryabi R, Kaveh MH. Effect of health education based on the protection motivation theory on malaria preventive behaviors in rural households of Kerman. Iran Int J Prev Med. 2014;5(4):463. 24829734 PMC4018595

[37] Chotiyaputta A. The Effectiveness of Health Education Program on Malaria Prevention among Junior High School Male Students in Sangklaburi District of Kanchanaburi Province. Mahidol University; 2001.

[38] Nushin P. Investigating the relationship between perceived self-efficacy and fertility behaviors in Iranian women covered by health centers in Mashhad city in order to reduce unwanted pregnancies. Fertility Infertility Q. 2008;8(30):91–78.

[39] Wurtele SK, Maddux JE. Relative contributions of protection motivation theory components in predicting exercise intentions and behavior. Health Psychol. 1987;6(5):453. 3678171 10.1037//0278-6133.6.5.453

[40] Courneya KS, Hellsten L-A. Cancer prevention as a source of exercise motivation: an experimental test using protection motivation theory. Psychol Health Med. 2001;6(1):59–64.

[41] Maseudi GR, Hosseini E-O, Mirzaei R, Shahrakipour M, Hosseini SA. The effect of education based on protection motivation theory on the harmful effects of solar rays on male students. Iran J Health Educ Health Promotion. 2015;2(4):322–30.

[42] Fakari FR, Simbar M. Coronavirus pandemic and worries during pregnancy; a letter to editor. Archives Acad Emerg Med. 2020;8(1). PMC7075675 32185371

[43] Rangarz Jeddy M, Eskandari N, Mohammadbeigi A, Gharlipour Z. Educational effect of applying health belief model on promoting preventive behaviors against COVID-19 in pregnant women. Health Educ Health Promotion. 2022;10(1):155–9.

[44] Karimy M, Gallali M, Niknami S, Aminshokravi F, Tavafian S. The effect of health education program based on Health Belief Model on the performance of pap smear test among women referring to health care centers in Zarandieh. J Jahrom Univ Med Sci. 2012;10(1).

[45] Saffari M, Shojaeizadeh D, Ghofranipour F, Heydarnia A, Pakpour A. Health education & promotion-theories, models & methods. Tehran: Sobhan Pub. 2009;55:59.

[46] Gholamreza S. Research in health education. Athar Sobahan Publishing House, Tehran. 2010;1:12–21.

[47] Walker K, Lynch M. Contributions of Anopheles larval control to malaria suppression in tropical Africa: review of achievements and potential. Med Vet Entomol. 2007;21(1):2–21. 17373942 10.1111/j.1365-2915.2007.00674.x

[48] Ng’ang’a PN, Shililu J, Jayasinghe G, Kimani V, Kabutha C, Kabuage L, et al. Malaria vector control practices in an irrigated rice agro-ecosystem in central Kenya and implications for malaria control. Malar J. 2008;7:1–9. 18667091 10.1186/1475-2875-7-146 PMC2517075

