# Supplementary material for: Effect of an educational intervention based on health belief model on preventive behaviors against malaria in over 18-year-old Afghan immigrants living in Parsian
Source: BMC Infect Dis. 2024 Oct 3;24:1101. doi: 10.1186/s12879-024-10016-9 (PMC11451138; doi:10.1186/s12879-024-10016-9)
Supplement: Supplementary file 1 — Supplementary Material 1 [file 12879_2024_10016_MOESM1_ESM.docx]

In the name of God

Questionnaire no.: ……………

This questionnaire aims to test your knowledge and beliefs about malaria. Please answer the questions completely and carefully, so that the answers reflect the facts and indicate your personal opinion. In addition, the content of the questionnaire will be considered completely confidential. We sincerely thank you for cooperation in this project.

**Knowledge:**

| # | Item | True | False | Don’t know |
| --- | --- | --- | --- | --- |
| 1 | Malaria can lead to brain damage and seizures. |  |  |  |
| 2 | Malaria is transmitted through the bite of the Anopheles mosquito. |  |  |  |
| 3 | Contaminated water can help transmit malaria. |  |  |  |
| 4 | Malaria does not afflict children. |  |  |  |
| 5 | There is no need to treat malaria. |  |  |  |
| 6 | A mosquito net should be used while resting or sleeping in the yard. |  |  |  |
| 7 | The female Anopheles mosquito is only found outdoors. |  |  |  |
| 8 | Malaria may cause fever and chills. |  |  |  |
| 9 | There is no need to dry still water to prevent malaria. |  |  |  |
| 10 | Malaria is caused by a microscopic parasite. |  |  |  |
| 11 | Long-sleeved clothes should be worn to prevent malaria. |  |  |  |

**Perceived susceptibility:**

| # | Item | Strongly agree | Agree | Undecided | Disagree | Strongly disagree |
| --- | --- | --- | --- | --- | --- | --- |
| 1 | I may also get afflicted with malaria. |  |  |  |  |  |
| 2 | I am concerned about getting afflcited with malaria. |  |  |  |  |  |
| 3 | I have lower chances of getting afflicted with malaria than people of my age. |  |  |  |  |  |
| 4 | Through traveling to Afghanistan, I may get afflcietd with malaria. |  |  |  |  |  |

**Perceived severity:**

| # | Item | Strongly agree | Agree | Undecided | Disagree | Strongly disagree |
| --- | --- | --- | --- | --- | --- | --- |
| 1 | I may die if I get afflicted with malaria. |  |  |  |  |  |
| 2 | Malaria imposes heavy medical expenses on me and my family. |  |  |  |  |  |
| 3 | It scares me to think about malaria. |  |  |  |  |  |
| 4 | I may get disabled if I am afflicted with malaria. |  |  |  |  |  |
| 5 | Malaria affects my social life and work. |  |  |  |  |  |
| 6 | The problems I will experience with malaria will last for long. |  |  |  |  |  |

**Perceived benefits:**

| # | Item | Strongly agree | Agree | Undecided | Disagree | Strongly disagree |
| --- | --- | --- | --- | --- | --- | --- |
| 1 | Malaria test can help diagnose the disease at an early stage. |  |  |  |  |  |
| 2 | A net at night helps prevent the malaria bite and disease. |  |  |  |  |  |
| 3 | Repellents help prevent the Anopheles mosquito bites. |  |  |  |  |  |
| 4 | Drying still water where the mosquito may reside kills Anopheles mosquito larvae. |  |  |  |  |  |
| 5 | Wearing long sleeves at night protects me from Anopheles mosquito bites. |  |  |  |  |  |
| 6 | Using a net on the door and window of the house prevents the Anopheles mosquito from entering. |  |  |  |  |  |

**Perceived barriers:**

| # | Item | Strongly agree | Agree | Undecided | Disagree | Strongly disagree |
| --- | --- | --- | --- | --- | --- | --- |
| 1 | I don’t have enough free time to do the mosquito infection test. |  |  |  |  |  |
| 2 | It is hard for me to control still water nearby my house. |  |  |  |  |  |
| 3 | Wearing long slleves at work does not let me work properly. |  |  |  |  |  |
| 4 | Going to the test lab is hard for me. |  |  |  |  |  |
| 5 | I forget to use insect repellent at night. |  |  |  |  |  |
| 6 | I cannot afford buying a mosquito net. |  |  |  |  |  |
| 7 | Using a moquito net is costly for me and my family. |  |  |  |  |  |
| 8 | I do not know how to use a mosquito net. |  |  |  |  |  |
| 9 | Using mosquito repellents does not affect whether I get infected or not. |  |  |  |  |  |

**Cues to action:**

| # | Item | Strongly agree | Agree | Undecided | Disagree | Strongly disagree |
| --- | --- | --- | --- | --- | --- | --- |
| 1 | Healthcare advice on malaria test encourages me to go for it. |  |  |  |  |  |
| 2 | Because a relative is already afflicted with Malaria, I will go for the test. |  |  |  |  |  |
| 3 | Doctor’s advice for the Malaria test encourages me to go for it. |  |  |  |  |  |
| 4 | If I have fever and chills, I will go for the Malaria test. |  |  |  |  |  |
| 5 | When I see or hear health messages about malaria, I am motivated to show malaria preventive behaviors. |  |  |  |  |  |
| 6 | Friends’ advice on using a mosquito net encourages me o go for it. |  |  |  |  |  |

**Perceived self-efficacy:**

| # | | Item | Always | Most often | Sometimes | Hardly ever | Never |
| --- | --- | --- | --- | --- | --- | --- | --- |
| 1 | I can use insect repellants to prevent anopheles bites at night. | |  |  |  |  |  |
| 2 | I can help the health staff take samples to diagnose malaria. | |  |  |  |  |  |
| 3 | I can easily visit the nearest health center for an examination to diagnose malaria. | |  |  |  |  |  |
| 4 | I can use the mosquito net to appropriately prevent the malaria. | |  |  |  |  |  |
| 5 | I am able to use net at doors and windows. | |  |  |  |  |  |
| 6 | I can easily remove the still water near my house or workplace. | |  |  |  |  |  |

**Behavior:**

| # | Item | Always | Most often | Sometimes | Hardly ever | Never |
| --- | --- | --- | --- | --- | --- | --- |
| 1 | I use insect repellants to prevent Anopheles mosquito bites. |  |  |  |  |  |
| 2 | To prevent malaria, at home, I use mosquito net at the doors and windows. |  |  |  |  |  |
| 3 | I wear long sleeves to prevent Anopheles bites. |  |  |  |  |  |
| 4 | I remove still water near my place of residence. |  |  |  |  |  |
| 5 | At night, I use appropriate mosquito nets while resting in open space. |  |  |  |  |  |
| 6 | In case there is a suspicous symptom, I tend to go for a malaria test. |  |  |  |  |  |
